# P-1386. Real-World Safety Profile of Pretomanid: An Analysis of FAERS Data

**DOI:** 10.1093/ofid/ofaf695.1573

**Published:** 2026-01-11

**Authors:** Hassaan Ali Ahmad

**Affiliations:** Faisalabad Medical University, Faisalabad, Punjab, Pakistan

## Abstract

**Background:**

Pretomanid received approval in the United States in 2019 as part of a combination regimen for the treatment of extensively drug-resistant and treatment-intolerant or non-responsive multidrug-resistant tuberculosis. Although clinical trials demonstrated efficacy, post-marketing safety data remain limited. This study examines real-world adverse events associated with pretomanid, using reports submitted to the FDA Adverse Event Reporting System (FAERS).

**Methods:**

The FAERS database was searched for all adverse events reported for Pretomanid from its approval through April 30, 2025. All adverse events reported for pretomanid were analyzed.

**Results:**

A total of 447 adverse event cases involving pretomanid were identified in the FAERS database. Most reports, 88.6 percent, were submitted between 2022 and 2025, with the highest number in 2024 (36.7 percent). Reports were mainly submitted by healthcare professionals (96.4 percent). Age data showed that 63.1 percent of cases involved individuals aged 18 to 64 years, 16.8 percent were between 65 and 85 years, and 2.9 percent were over 85. Pediatric patients made up 2.5 percent. Regarding sex, 58.2 percent of patients were male, 33.3 percent female, and 8.5 percent unspecified. A total of 72 cases, or 16.1 percent, listed death as the outcome. The most frequently reported events were death (16.1 percent), anaemia (8.7 percent), QT prolongation (6.7 percent), nausea (6.5 percent), acute kidney injury (5.6 percent), off-label use (4.9 percent), neuropathy peripheral (4.9 percent), hepatotoxicity (4.9 percent), alanine aminotransferase increased (4.3 percent), and aspartate aminotransferase increased (3.8 percent). Additional events included cardiac failure, dyspnoea, decreased appetite, respiratory failure, vomiting, diarrhoea, pancreatitis, cardiac failure acute, and cardiopulmonary failure.

**Conclusion:**

Real-world data from FAERS indicate that pretomanid is associated with a substantial proportion of serious and potentially fatal adverse events. QTc prolongation, hematologic toxicity, and renal impairment are key safety concerns. Ongoing pharmacovigilance is critical to ensure the safe use of pretomanid, especially in older adults and complex clinical settings.

**Disclosures:**

All Authors: No reported disclosures

